# Hearing loss and small and large fibre neuropathy associated with the heterozygous variants c.20A>T in HBB and del-3.7 in HBA

**DOI:** 10.1016/j.clinsp.2025.100597

**Published:** 2025-02-28

**Authors:** Josef Finsterer, Fulvio A. Scorza, Carla A. Scorza, Ana C. Fiorini

**Affiliations:** a Neurology and Neurophysiology Center, Vienna, Austria; b Escola Paulista de Medicina/Universidade Federal de São Paulo (UNIFESP/EPM), São Paulo, SP, Brazil; c Programa de Estudos Pós-Graduado em Fonoaudiologia, Pontifícia Universidade Católica de São Paulo (PUC-SP), São Paulo, SP, Brazil; d Departamento de Fonoaudiologia, Escola Paulista de Medicina/Universidade Federal de São Paulo (EPM/UNIFESP), São Paulo, SP, Brazil

## Correspondence

Hemoglobinopathies are genetic diseases that are among the most common hereditary diseases worldwide.^1^ Nowadays, they are also becoming increasingly common in the Western world due to immigration.^1^ They can be divided into two main groups: Thalassemia syndromes and structural hemoglobin variants (abnormal hemoglobins).^1^ α- and β-thalassemia are the main types of thalassemia syndromes.^1^ The main structural hemoglobin variants are HbS, HbE and HbC.^1^ There are numerous subtypes and combined types in each group.^1^ α-thalassemia results from a defect in the synthesis of the α-globin chain, often caused by deletions of one or both α-genes (HBA) on the same allele.^2^ One of these deletions causing α-thalassemia is the 3.7kb deletion in HBA.^2^ The prevalence of heterozygous 3.7 kb HBA deletion in α-thalassemia patients has been calculated to be 11 %.^2^

Hemoglobin S (HbS), also known as sickle hemoglobin, is a structural variant of normal adult β-hemoglobin (HbB) caused by an amino acid substitution at position 6 of the β-Globin chain (c.20A>T; p.Glu6-Val).^3^ Heterozygotes of the mutation are almost always asymptomatic (HBS), while homozygotes (HbSS) suffer from sickle cell anemia, which often leads to acute and chronic complications such as vaso-occlusive, acute chest or hemolytic crisis.^3^

Patients with thalassemia are known to complain of numbness and weakness of the lower extremities.^4^ Up to 80 % of patients with thalassemia develop mild sensory polyneuropathy.^4^ While neuropathy has rarely been reported in α-thalassemia, neuropathy is more common in patients with β-thalassemia.^4^, ^5^, ^6^ Hearing loss has been reported in patients with both α- and β-thalassemia.^7^^,^^8^ An adult patient carrying a mutation in HBA and HBB and phenotypically manifesting with hearing loss and Small Fiber Neuropathy (SFN) that progresses to Large Fiber Neuropathy (LFN) has not been reported to date.

The patient is a 48-year-old African man, 170 cm tall, weighing 75 kg, who developed burning and tingling paresthesias on the soles of his feet 14-years before presentation, which later spread over the feet to the lateral parts of the lower legs, with the right side clearly dominating. The medical history included recurrent malaria tropical as a child in Gambia, detection of the heterozygous variants del-3.7 in HBA and c.20A>T in HBB at the age of 39, depression at the age of 46 to 47 years, which required antidepressant treatment and psychotherapy, a mild COVID-19 infection and a sensorineural hearing loss that had been treated with hearing aids since the age of 43. There was no evidence of hearing loss or a neuromuscular disorder in his family history. However, there was a suspicion of consanguinity between the paternal grandfather and the maternal grandfather.

Clinical neurologic examination revealed bilateral hypoacusis, decreased tendon reflexes in the upper limbs, absent tendon reflexes in the lower limbs, a mute sole on both sides, dysesthesias and paresthesias in the soles, feet, and lateral lower limbs, stocking-like hypoesthesia for touch and vibration, unsteady walking on a line, and a tendency to fall when walking blindly. Blood tests revealed a creatine kinase of 330U/L (n, < 171U/L) and decreased HDL, but were otherwise negative for any cause of acquired polyneuropathy. Nerve Conduction Studies (NCS) at 40-years of age were normal. Extensive investigations including lumbar puncture for causes of polyneuropathy were repeatedly inconclusive. However, a skin biopsy from the thigh and lower leg revealed a marked reduction in intraepidermal nerve fiber density in the lower limb (mean 4.4±0.69 [n, 9.8 ± 0.5]) and thigh (mean 8.33 ± 2 [n, 21.0 ± 10.4]), which is why SFN was diagnosed. Treatment with glucocorticoids and Intravenous Immunoglobulins (IVIGs) was ineffective. As duloxetine, quetiapine, sertraline and pregabalin at high doses were only marginally helpful, the patient discontinued these medications. NCS at 48-years of age revealed marked axonal sensorimotor LFN. PGB was started again. He had never needed blood transfusions before.

The presented patient is interesting in several respects. First, he is the first case with SFN and LFN due to a heterozygous mutation in HBA and HBB. Secondly, the patient initially showed phenotypic SFN, which later developed into LFN. The latency period after which SFN turned into a combination of SFN and LFN was about 14-years. Third, SFN symptoms did not respond to glucocorticoids or IVIGs, suggesting that SFN is not due to immune-mediated pathophysiology, but rather is genetically determined.

In favor of a causal relationship between hemoglobinopathy and SFN/LFN is the fact that such a relationship has already been reported several times in HBB mutation carriers, that the immunosuppressive treatment was ineffective, and that it is conceivable that mutated hemoglobin is neurotoxic. However, the fact that the specific genetic constellation of the index patient has not previously been reported in association with SFN/LFN, that other types of hereditary neuropathy have not been ruled out, that the patient never had anemia, and that the pathophysiologic mechanism by which hemoglobinopathy could damage the peripheral nerve is not fully understood argues against a causal relationship. However, it is speculated that hemoglobin and its degradation products (heme, bilirubin, free iron) outside the controlled environment of the erythrocyte via the iron atom are involved in toxic redox reactions that lead to oxidation of DNA, proteins, and lipids, resulting in cellular dysfunction and nerve damage.^9^ It is possible that these effects are exacerbated by mutated hemoglobins. There is evidence that haptoglobin can antagonize the neurotoxic effect of hemoglobin.^9^

In summary, this case shows that the heterozygous mutation c.20A>T in HBB together with the deletion -3.7 in HBA can manifest phenotypically without anemia, but initially with SFN, which develops into LFN as the disease progresses, and with hearing loss. The neuropathy may manifest primarily as painful dysesthesia, paresthesia, pallhypesthesia, and gait disturbance. These patients may benefit from hearing aids and symptomatic treatment of neuropathic pain.

## Authors’ contributions

JF: Conceptualization, Formal analysis, Investigation, - Validation, Writing - original draft, - Writing - review and editing. CS, FS, AF: supervision, valudation, writing- review and editing.

## Compliance with ethics guidelines

This article is based on previously conducted studies and does not contain any new studies with human participants or animals performed by any of the authors.

## Data access statement

All data are available from the corresponding author.

## Ethics statement

Not applicable.

## Funding

No funding was received.

## Declaration of competing interest

The author declares that the research was conducted in the absence of any commercial or financial relationships that could be construed as a potential conflict of interest.
